# Hydrogen-Rich Saline Activated Autophagy via HIF-1*α* Pathways in Neuropathic Pain Model

**DOI:** 10.1155/2018/4670834

**Published:** 2018-05-17

**Authors:** Huixing Wang, Xiaodong Huo, Hongguang Chen, Bo Li, Jingzhi Liu, Wenting Ma, Xiaojuan Wang, Keliang Xie, Yonghao Yu, Kemei Shi

**Affiliations:** ^1^Pain Management Center, Second Hospital of Tianjin Medical University, Tianjin 300211, China; ^2^Central Laboratory, Tianjin Research Institute of Urology, The Second Hospital of Tianjin Medical University, Tianjin 300211, China; ^3^Department of Oncology, Second Hospital of Tianjin Medical University, Tianjin 300211, China; ^4^Department of Anesthesiology, Tianjin Medical University General Hospital, Tianjin Research Institute of Anesthesiology, Tianjin 300211, China

## Abstract

**Background:**

Neuropathic pain is a chronic and intractable pain, with very few effective analgesics. It involves an impaired cell autophagy process. Hydrogen-rich saline (HRS) reportedly reduces allodynia and hyperalgesia in a neuropathic pain model; however, it is unknown whether these effects involve autophagy induction.

**Methods:**

We investigated the relationship between HRS and cell autophagy in a neuropathic pain model generated by chronic constriction injury (CCI) in Sprague–Dawley rats. Rats received an intraperitoneal injection of HRS (10 mL/kg daily, from 1 day before until 14 days after CCI), 3MA (autophagy inhibitor), 2ME2 (HIF-1*α* inhibitor), or EDHB (HIF-1*α* agonist). The mechanical withdrawal threshold (MWT) and thermal withdrawal latency (TWL) were tested 1 day before and 1, 3, 7, 10, and 14 days after the operation. HIF-1*α* and cell autophagy markers in the spinal cord were evaluated by western blotting and real-time PCR assays at 14 days after CCI. Autophagosomes with double membranes were identified by transmission electron microscopy.

**Results:**

CCI caused behavioral hypersensitivity to mechanical and thermal stimulation in the hind-paw of the injured side. HRS improved MWT and TWL, activated autophagy, and increased autophagosomes and autolysosomes in CCI rats. 3-MA aggravated hyperalgesia and allodynia and suppressed autophagy, while EDHB attenuated hyperalgesia and activated the autophagy procedure and the HIF-1*α* downstream target gene BNIP3. HIF-1*α* inhibitors reversed the regulatory effects of HRS on autophagy in CCI rats at 14 days after spinal cord injury.

**Conclusion:**

HRS reduced mechanical hyperalgesia and activation of cell autophagy in neuropathic pain through a HIF1-dependent pathway.

## 1. Introduction

Neuropathic pain (NP) is a chronic and disabling pain caused by nervous system lesions or disease. It is characterized by allodynia, hyperalgesia, and spontaneous pain, which is only partly treated by the current limited therapies. According to statistics, the prevalence of NP is 5–7%, and millions of patients suffer from NP globally [1]. Long-term pain not only affects sleep quality but causes depression and mental disorder, and even suicidal tendencies, and thus severely influences quality of life [2]. However, the specific mechanism of NP is unclear, and the treatment of NP remains a thorny problem.

Hydrogen is a colorless, odorless, antioxidant diatomic gas. Dole and colleagues first reported the therapeutic effect of hydrogen in cancer animal experiments in 1975 [3]. After Ohsawa et al. [4] published an article showing that inhalation of 1–4% hydrogen could significantly improve cerebral ischemia-reperfusion injury, an effect that may have been associated with a selective antioxidant effect, the use of hydrogen gained more interest. Recent studies have revealed that hydrogen gas or hydrogen-rich saline (HRS) has beneficial effects in a variety of diseases, due to its anti-inflammatory, antioxidative, and antiapoptotic effects [5, 6]. Ge et al. have reported that intrathecal infusion of HRS reversed the overexpression of MnSOD nitrated at tyrosine after ligation of the spinal nerve at L5, decreased the activation of astrocytes and microglia, and attenuated the expression of interleukin-1*β* (IL-1*β*) and tumor necrosis factor-*α* (TNF-*α*) in the spinal cord [7]. We have shown that administration of HRS alleviates mechanical hyperalgesia and thermal hyperalgesia after remifentanil administration [8] as well as in a CCI model [9], via an anti-inflammatory and antioxidative effect. However, the molecular mechanism by which HRS affects NP remains elusive.

Autophagy is a crucial homeostatic mechanism that alleviates metabolic stress and degradation within the cytoplasm and cell organelles [10]. During this process, dysfunctional cytosolic components, such as protein and RNA, are degraded via the autophagosome. If cellular responses caused by stress were switched from apoptosis to autophagic responses, cells may survive [11]. There are three types of autophagy: macroautophagy, microautophagy, and chaperone-mediated autophagy. Many studies have indicated that autophagic dysfunction is involved in many conditions, such as neurodegenerative diseases and cancer [12, 13]. In the early stages of such diseases, appropriate activation of autophagy can remove abnormal proteins and damaged organelles, while an antioxidant can slow down the progress of neurodegeneration [14] and is thus a promising therapeutic strategy.

Recently, a number of publications have confirmed that cell autophagy is involved in the occurrence and development of NP. Berliocchi et al. [15] showed that expression of the autophagy marker protein LC3 and autophagic degradation protein P62 were increased in the spinal dorsal horn of NP model rats and concluded that lysosomal degradation or autophagy is impaired in NP, as also supported in another NP model [16]. Feng et al. [17] confirmed that activation autophagy in the spinal microglia can relieve pain after intrathecal injection of rapamycin (a common autophagy revulsant) in a spinal nerve ligation (SNL) model, which indicated that early induction of autophagy can relieve the pain in NP.

It has been reported that HRS is able to attenuate acute kidney injury after liver transplantation, partly by reducing apoptosis and modulating p53-mediated autophagy [18]. HRS has a neuroprotective effect against hypoxia-ischemia-induced neonatal brain injury in mice, mediated in part by reducing endoplasmic reticulum stress and increasing autophagy [19]. However, whether HRS can be used to treat NP by regulating cell autophagy has not been investigated.

Hypoxia-inducible factor-1*α* (HIF-1*α*) is a regulated subunit of hypoxia-inducible factor 1 (HIF-1), a transcription factor that plays a pivotal role in regulating gene expression to maintain oxygen homeostasis [20]. HIF-1 is composed of HIF-1*α* and HIF-1*β* subunits. Under hypoxic and nonhypoxic stimuli, such as cytokines, free radicals, growth factors, and hormone stimulation, HIF-1*α* hydroxylation is inhibited, allowing its translocation into the nucleus, where it binds to HIF-1*β* to form an active HIF-1 complex. HIF-1 then initiates the transcription of an array of target genes that are vital for cellular adaption to hypoxic and nonhypoxic stimuli. Hypoxia influences autophagy in part via the activation of the HIF-dependent pathways [21]. HIF-1*α* plays an important role in the induction of autophagy via the inhibition of the mammalian target of rapamycin (mTOR) [22] or via enhanced expression of its target gene,* BNIP3*, and disruption of the Bcl-2-Beclin-1 complex [23]. HIF-1*α* has a neuroprotective effect by inducing autophagy in ischemic brain injury [24, 25]

We hypothesized that HRS treatment could alleviate allodynia and hyperalgesia and activate cell autophagy in NP through a HIF-1-dependent pathway.

## 2. Materials and Methods

### 2.1. Animals

The experiments were performed on male Sprague–Dawley rats, weighing 180–220 g. All animals were provided by the Military Academy of Medical Sciences Animal Laboratory Center, Tianjin, China, and were housed in a temperature-controlled (25°C) room, with a 12 h light-dark cycle, and were given free access to food and water. All animal care and experimental procedures were strictly under obligations of Institutional Animal Care and Use Committee of the Second Hospital of Tianjin Medical University. All efforts were made to minimize suffering.

### 2.2. HRS Production

HRS was prepared by a hydrogen-rich water producing apparatus (YUTAKA Engineering Co., Tokyo, Japan). Hydrogen was dissolved in normal saline under high pressure (0.4 MPa) for 6 hours to reach a supersaturated level. The saturated HRS was stored under atmospheric pressure in an aluminum bag at 4°C, sterilized by *γ* radiation. In order to ensure a constant concentration more than 0.6 mmol/L, it should be prepared freshly every week.

### 2.3. Experimental Protocol

This study was divided into three parts. In Experiment 1, we explored changes in the behavior and the influence of cell autophagy after intraperitoneal HRS injection for NP in the rat model. In Experiment 2, we researched autophagy activation in the CCI model after HRS injection and administration of an autophagy inhibitor. In Experiment 3, we observed whether the effects of HRS on autophagy in NP model rats were associated with the HIF-1 pathway.

**Experiment 1 .**

Thirty-six SD rats were randomly divided into three groups (*n* = 12 per group): group S (the sham group), group C (the CCI group), and group C + H (the CCI + HRS treatment group). HRS was injected intraperitoneally at a daily dose of 10 mL/kg for 14 days after CCI surgery. In group S, physiological saline was injected in the same manner. MWT and TWL were measured at 1 d before and 1, 3, 7, 10, and 14 days after surgery. The L4–L6 segments of the spinal cord were collected at 14 days after surgery to determine autophagy-related protein and mRNA (Beclin-1, P62, and HIF-1*α*) expression.

**Experiment 2 .**

Sixty SD rats were randomly divided into five groups (*n* = 12 per group): group S (the sham group), group C (the CCI group), group C + H (the CCI + HRS treatment group), group C + M (CCI + 3-MA treatment group), and group C + H + M (CCI + HRS + 3-MA treatment group). HRS and physiologic saline were injected as in Experiment 1. 3-MA, a common autophagy inhibitor, was injected intraperitoneally (10 mg/kg), 1 h after surgery and daily thereafter for 14 days. MWT and TWL were measured at 1 d before and at 1, 3, 7, 10, and 14 days after surgery. The L4–L6 segments of the spinal cord were collected at 14 days after surgery, to determine autophagy-related protein expression (Beclin-1 and P62). Autophagosomes, with double membranes, were identified morphologically by transmission electron microscopy (TEM).

**Experiment 3 .**

A total of 96 rats were randomly divided into eight groups (*n* = 12 per group): group S, group S + H, group C, group C + H, group C + 2Me2, group C + H + 2Me2, group C + EDHB, and group C + H + EDHB. The HRS and physiologic saline were injected as in Experiment 1. According to a previously published report [26], 2ME2 was dissolved in 0.5% dimethylsulfoxide, and a dose of 10 mg/kg was injected intraperitoneally 30 min after CCI. As reported previously [27], EDHB, a prolyl hydroxylase domain protein inhibitor, and HIF-1*α* agonist with EDHB (100 mg/kg, intraperitoneally) were injected 30 min after CCI. The MWT and TWL were tested 1 day before surgery and daily thereafter for 14 days. The L4–L6 segments of the spinal cord were collected after behavioral testing to determine autophagy-related protein and mRNA expression (Beclin-1, HIF-1*α*, and BNIP3). Autophagosomes, with double membranes, were also identified morphologically by transmission electron microscopy (TEM).

### 2.4. Neuropathic Pain Model

According to the methods described by Bennett and Xie [26], an NP model was generated by chronic constriction injury (CCI). Rats were anesthetized by intraperitoneal injection of 10% chloral hydrate (300 mg/kg); the skin was sterilized, and the skin and muscular tissue of the left biceps femoris were incised layer by layer, to expose the sciatic nerve. An 8 mm portion of the sciatic nerve was freed from the adhering tissue at the sciatic trifurcation, and the sciatic nerve trunk was ligated in four places with 4.0 silk; the ligatures were tied loosely at 1 mm intervals. The wound was then flushed with physiological saline, the local skin area was disinfected with povidone-iodine solution, and the leg was sutured layer by layer. Postoperatively, the rats were kept in a quiet and warm single cage. For sham-operated rats, the same process was performed, without ligation of the nerve.

### 2.5. Behavior Tests

Before behavioral tests, rats were taken to the behavior room for 30 min to adapt to the environment. The mechanical withdrawal threshold (MWT) and thermal withdrawal latency (TWL) were tested at 1 day before and 1, 3, 7, 10, and 14 days after surgery.

For the MWT, the rats were acclimatized to a mesh cage plane for 15 min at first. Then, we used a handheld electronic Von Frey pain measurement instrument (Bio EVF3, Bioseb Ltd., Vitrolles, France) to test for MWT of the ipsilateral paw. Every rat was subjected to three tests, with 15 min tests between intervals, and the average was taken as the MWT. To prevent injury to the rats' feet, the maximum pressure was set at 60 g; if a rat showed no reaction, the rat was excluded from the experiment.

To measure TWL, a hot plate (YLS-6B, Shanghai Precision Instrument Co., Ltd., Shanghai, China) was set to 52 ± 0.2°C. The intensity of the thermal stimulation was kept constant throughout the experiment. The reaction time of the rats to the heat stimulation of the hind leg was measured; reactions included shrinking away, licking the foot, or squeaking. Every rat was subjected to three tests, with an interval of 10 min between tests, and the average was taken as the TWL. To prevent scalding the rats, the time limit was set to 60 s.

### 2.6. Western Blot

After 14 days, the animals were anesthetized with chloral hydrate (10%) after the last behavioral test. The animals were sacrificed after rapid 150 mL 0.9% saline heart perfusion. The L4–L6 spinal cord samples were removed rapidly and homogenized in cold lysis buffer containing protease inhibitor cocktail (Sigma-Aldrich, St Louis, MO, USA) and centrifuged at 10,000 ×g for 20 min at 4°C. The supernatant was removed as the total protein, and protein concentration was tested using a BCA protein assay kit (Beyotime Institute of Biotechnology, Jiangsu, China). Thereafter, the supernatant was mixed with loading buffer and heated at 100°C for 5 min. Samples were electrophoresed on 8–12% SDS-PAGE gels, transferred to PVDF membranes, blocked in 5% defatted milk for 1 h, and incubated with primary antibodies against Beclin-1 (1 : 500, Abcam, Cambridge, UK), P62 (1 : 1000, Abcam, Cambridge, UK), HIF-1*α* (1 : 1000, Abcam, UK), BNIP3 (1 : 600, Abcam, UK), and *β*-actin (1 : 4000, Abcam, UK) at 4°C overnight. The membranes were then washed and incubated with HRP-conjugated goat anti-rabbit IgG secondary antibodies (1 : 1000, Abcam, UK) for 1 h at room temperature. Immunoblots were visualized by ECL chemiluminescence imaging system and photographed for quantitative analysis by using the Quantity One software (BIO-RAD, Tokyo, Japan).

### 2.7. Real-Time PCR

At 14 days after surgery and after behavioral testing, animals were anesthetized with chloral hydrate (10%) and sacrificed after rapid heart perfusion with 150 mL 0.9% saline. The spinal cord samples were collected and stored at −80°C for detection of Beclin-1, P62, and HIF-1*α* mRNA expression. Real-time PCR (RT-PCR) was performed as per the manufacturer's instructions. Total mRNA was extracted using TRIzol reagent (Invitrogen, Carlsbad, CA, USA). Reverse transcription was performed using a RETROscript kit (Ambion Inc., Austin, TX, USA). Amplification reactions were carried out using SYBRgreen polymerase chain reaction (PCR) master mix (Applied Biosystems, Foster City, CA, USA). The mRNA levels in the mRNA were determined using the Mastercycler ep realplex2 system (Eppendorf, Hamburg, Germany). The primer sequences were showed in Table 1. Values were normalized to those of GAPDH as housekeeping gene.

### 2.8. Electron Microscopy

Four rats in each group in Experiment 2 (*n* = 12 per group) were anesthetized with chloral hydrate (10%) after the last behavioral test. The L4–L6 spinal cord samples were removed rapidly after rapid heart perfusion with 150 mL 0.9% saline (containing 1% heparin) and fixed in a mixture of 4% paraformaldehyde and 2% glutaraldehyde solution. After 2 h, the samples were cut into small pieces (1 mm^3^) and rapidly placed in 3% glutaraldehyde for fixation for 2 h. Samples were then dehydrated in acetone and embedded in Araldite (Fluka, Buchs, Switzerland). Ultrathin sections were prepared and double-stained with uranyl acetate and lead citrate. Ultrastructural organelles were observed under TEM (JEM-1200X, Shimadzu, Japan). Ten fields of view were analyzed for every sample, and the numbers of autophagosomes and autolysosomes were recorded.

### 2.9. Statistical Analysis

All results were analyzed using SPSS 18.0 (SPSS; Chicago, IL, USA) and Prism® version 6 (GraphPad, La Jolla, CA, USA). Statistical differences among groups were analyzed with one-way analysis of variance (ANOVA), followed by Tukey's test. All data were expressed as mean ± SD. Statistical significance was defined as *P* < 0.05.

## 3. Results

### 3.1. HRS Treatment Improves MWT and TWL in Rats with NP

**Figure 1:**
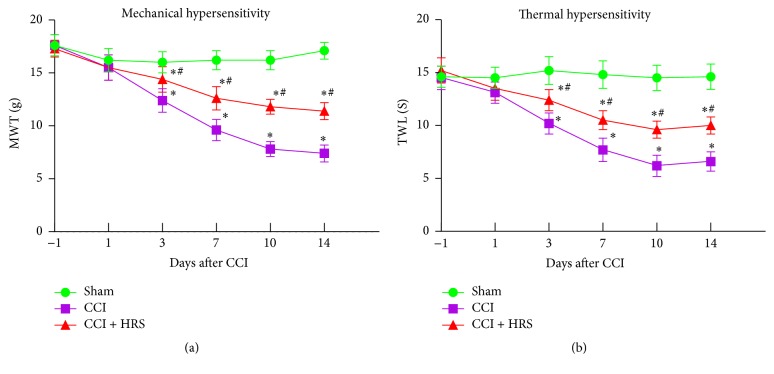
*Effect of HRS on thermal hypersensitivity and mechanical hypersensitivity induced by CCI rats 1 day before and 1, 3 7, 10, and 14 days after CCI.* (a) Mechanical withdrawal threshold. (b) Thermal withdrawal latency. Data are expressed as mean ± SD; ^*∗*^*P* < 0.05 versus sham; ^#^*P* < 0.05 versus CCI.

In our study, we measured MWT and TWL 1 day before and 1, 3, 7, 10, and 14 days after CCI. On preoperative day 1, the MWT and TWL of each group were not statistically significantly different (*P* > 0.05). Compared with the sham group, MWT and TWL decreased from day 3 to day 14 in group C (*P* < 0.05, *n* = 12, one-way ANOVA). Compared with group C, HRS treatment largely reversed the decrease in MWT and TWL in group C + H (*P* < 0.05, *n* = 12, one-way ANOVA; Figures 1(a) and 1(b)). Thus, HRS treatment showed a significant regulatory role in thermal hyperalgesia and mechanical allodynia.

### 3.2. HRS Treatment Activates Autophagy in Rats with CCI of the Spinal Cord

**Figure 2:**
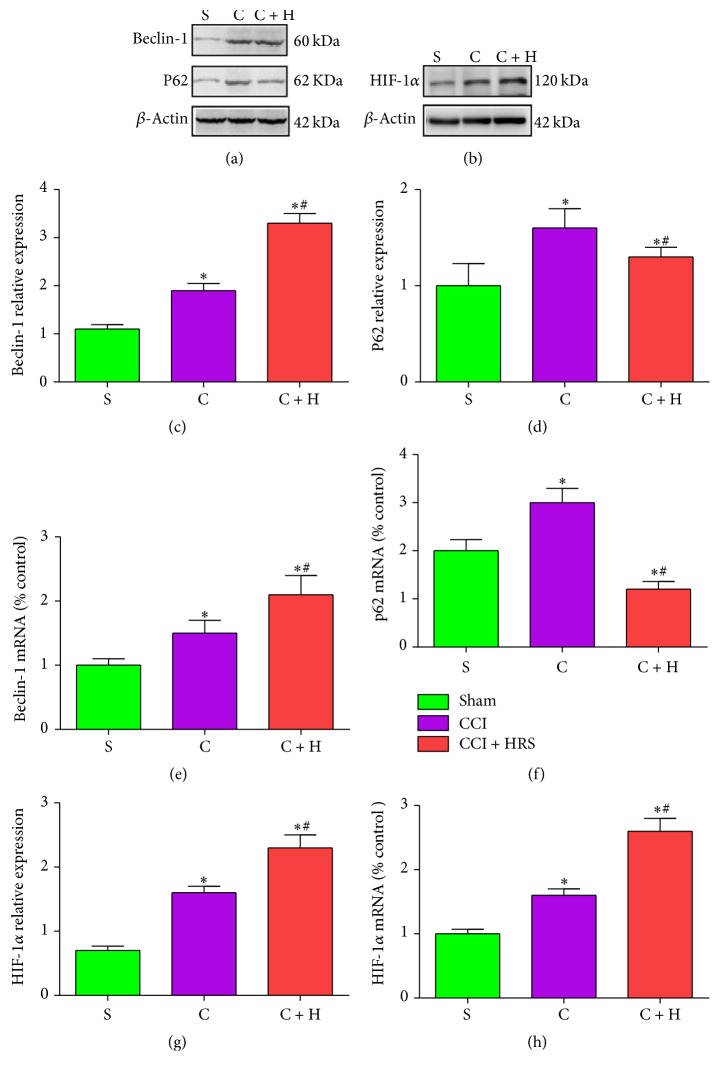
*The expression of autophagy associated protein mRNA and in three groups after HRS treatment.* ((a)-(b)) The western blot result of autophagy associated protein and HIF-1*α* in spinal cord at 14 days, respectively. ((c)-(d)) The expression of Beclin-1 and P62 protein in spinal cord at 14 days, respectively. ((e)-(f)) The mRNA expression of Beclin-1 and P62 in spinal cord at 14 days, respectively. (g) The expression of HIF-1*α* protein in spinal cord at 14 days. (h) The mRNA expression of HIF-1*α* in spinal cord at 14 days. Data are expressed as mean ± SD; ^*∗*^*P* < 0.05 versus sham; ^#^*P* < 0.05 versus CCI.

We investigated the protein and mRNA expression of autophagy markers Beclin-1 and P62 in the spinal cord of rats 14 days after nerve injury. Beclin-1 plays a role in the initiation step and is essential for the formation of the autophagosome [28]. In contrast to sham-operated rats, the mRNA and protein expressions of Beclin-1 in the spinal cord of group C were significantly increased on the 14th postoperative day (*P* < 0.05, *n* = 12, one-way ANOVA). Compared with that in CCI rats, Beclin-1 expression in the spinal cord of group C + H was significantly upregulated (Figures 2(a), 2(c), and 2(e)). Thus, HRS treatment increased expression of Beclin-1 in rats with CCI of the spinal cord.

However, the ubiquitin-binding protein P62/SQSTM1 is an autophagy substrate, which, upon direct binding to LC3, incorporates into autophagosomes and is efficiently degraded by autophagy [29, 30]. Thus, when autophagy is arrested, P62 accumulates [31]. Compared to group S, P62 in the spinal cord of group C was significantly increased on the 14th postoperative day (*P* < 0.05, *n* = 12, one-way ANOVA; Figures 2(a), 2(d), and 2(f)). Compared with that in CCI rats, P62 in the spinal cord of group C + H was significantly decreased (Figures 2(a), 2(d), and 2(f)). Thus, HRS treatment activated the autophagy pathway in rats with CCI of the spinal cord.

### 3.3. HRS Induces Upregulation of HIF-1*α* mRNA and Protein Expression in Rats with CCI of the Spinal Cord

In contrast to sham-operated rats, HIF-1*α* mRNA and protein expression in the spinal cord of group C were significantly increased on the 14th postoperative day (*P* < 0.05, *n* = 12, one-way ANOVA; Figures 2(b), 2(g), and 2(h)). Compared with that in CCI rats, the HIF-1*α* mRNA levels in the spinal cord of group C  +  H were significantly upregulated (Figures 2(b), 2(g) and 2(h)). Thus, HRS treatment increased HIF-1*α* expression in rats with CCI of the spinal cord.

### 3.4. Inhibition of Autophagy by 3-MA Aggravates Hyperalgesia and Allodynia in HRS-Treated CCI Rats

**Figure 3:**
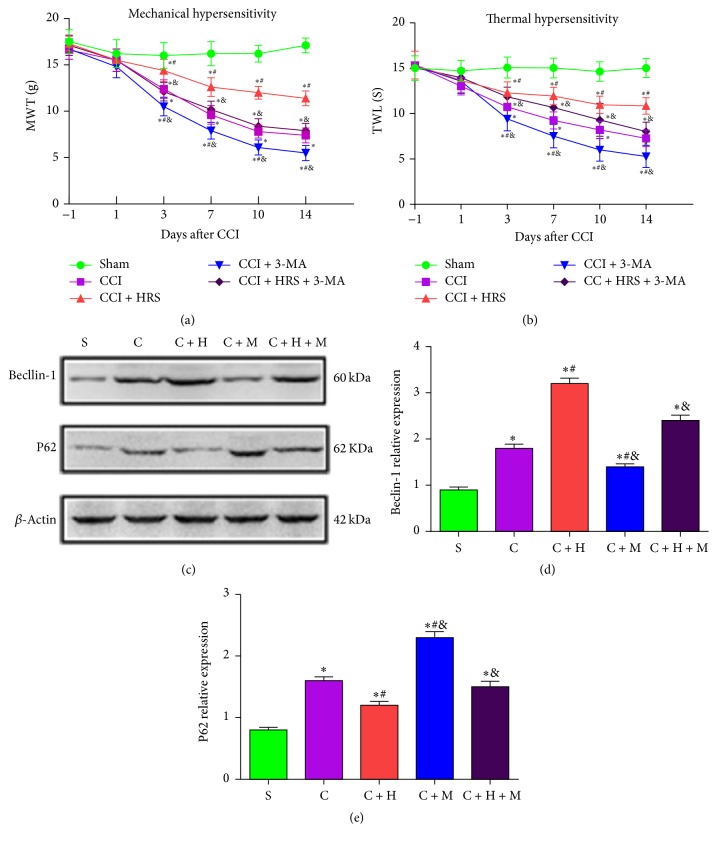
*Effect of 3-MA on thermal hypersensitivity and mechanical hypersensitivity 1 day before and 1, 3, 7, 10, and 14 days after CCI.* (a) Mechanical withdrawal threshold. (b) Thermal withdrawal latency. ((c)–(e))* Effect of 3-MA on the expression of autophagy associated protein Beclin-1 and P62 after HRS treatment, respectively.* Data are expressed as mean ± SD; ^*∗*^*P* < 0.05 versus sham; ^#^*P* < 0.05 versus CCI; ^&^*P* < 0.05 versus CCI + HRS.

We further researched autophagy activation in the CCI model after HRS injection by using an autophagy inhibitor. Compared with the baseline on preoperative day 1, the MWT and TWL of each group were not statistically significantly different (*P* > 0.05). Compared with the sham group, the MWT and TWL decreased from day 3 to day 14 in the other CCI groups: group C + M and group C + H + M (*P* < 0.05, *n* = 12, one-way ANOVA). Compared with group C, these abnormal changes were significantly alleviated by HRS treatment (*P* < 0.05). Group C + M showed statistically significantly reduced MWT and TWL values during postoperative days 3–14 (*P* < 0.05). There was no statistically significant difference in these values for group C + H + M (*P* > 0.05). Compared with group C + H, the MWT and TWL values were reduced statistically significantly in group C + M and group C + H + M, on postoperative days 3, 7, 10, and 14 (*P* < 0.05, *n* = 12, one-way ANOVA; Figures 3(a) and 3(b)). Thus, HRS treatment played a significant regulatory role in thermal hyperalgesia and mechanical allodynia, while the autophagy inhibitor 3-MA aggravated thermal hyperalgesia and mechanical allodynia from days 3 to 14 after CCI (Figures 3(a) and 3(b)).

### 3.5. 3-MA Suppresses Autophagy in HRS-Treated Rats with CCI of the Spinal Cord

We also investigated the mRNA and protein autophagy markers Beclin-1 and P62 in the spinal cord of mice at 14 days after nerve injury. The Beclin-1 levels in the spinal cord of groups with nerve-injury (group C, group C + H, group C + M. and group C + H + M) were significantly increased on the 14th postoperative day compared with that in sham-operated rats (*P* < 0.05, *n* = 12, one-way ANOVA; Figures 3(c) and 3(d)). In contrast, the P62 levels in the spinal cord of rats with nerve-injury (group C, group C + H, group C + M, and group C + H + M) were significantly decreased on the 14th postoperative day as compared with that in the sham-operated rats (*P* < 0.05, *n* = 12, one-way ANOVA; Figures 3(c) and 3(e)). Compared with that in CCI rats, Beclin-1 levels were significantly increased, and P62 levels were significantly decreased in the spinal cord of both groups C + H. Beclin-1 levels were also significantly decreased, and P62 levels were significantly increased (both *P* < 0.05) in the spinal cord of group C + M. Furthermore, compared with that in group C + H, the Beclin-1 levels in the spinal cord of group C + H + M were significantly decreased, and P62 levels were significantly increased (*P* < 0.05; Figures 3(c)–3(e)). Thus, HRS treatment activated the autophagy pathway in rats with CCI of the spinal cord. The autophagy inhibitor 3-MA suppressed autophagy in HRS-treated rats with CCI of the spinal cord.

### 3.6. HRS Treatment Increases Autophagosomes and Autolysosomes in Rats with CCI of the Spinal Cord

**Figure 4:**
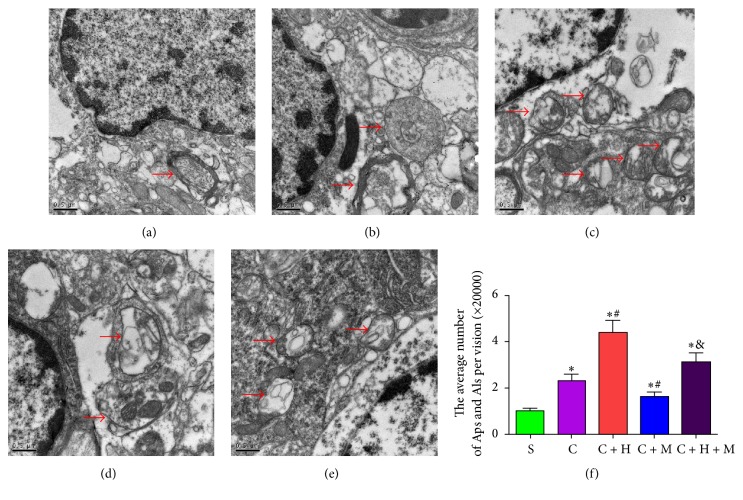
*Autophagosomes (Aps) and autolysosomes (Als) in the spinal cord neurons were evaluated with TEM.* (a) Sham group. (b) CCI group. (c) CCI + HRS. (d) CCI + 3MA group. (e) CCI + 3MA + HRS group. (f) The number of autophagosomes (Aps) and autolysosomes (Als) in the spinal cord; ^*∗*^*P* < 0.05 versus sham; ^#^*P* < 0.05 versus CCI; ^&^*P* < 0.05 versus CCI + HRS. One autophagosome has engulfed a mitochondrion (arrow). Scale Bar = 500 nm.

The number of autophagosomes and autolysosomes was evaluated with TEM. The autophagosomes with double membranes were identified morphologically by TEM [28]. The number of autophagosomes and autolysosomes in the spinal cord of nerve-injured rat groups (group C, group C + H, group C + M, and group C + H + M) were significantly increased on the 14th postoperative day compared with that in sham-operated rats (*P* < 0.05). Compared with group C and group S, HRS injection significantly increased the number of autophagosomes and autolysosomes in the spinal cord at 14 days after CCI (Figures 4(a)–4(f)), whereas administration of 3-MA significantly reduced the number of autophagosomes and autolysosomes in group C + M and group C + H + M (Figures 4(a)–4(f)).

### 3.7. EDHB and HRS Attenuate Hyperalgesia and Allodynia in CCI Rats at 14 Days

**Figure 5:**
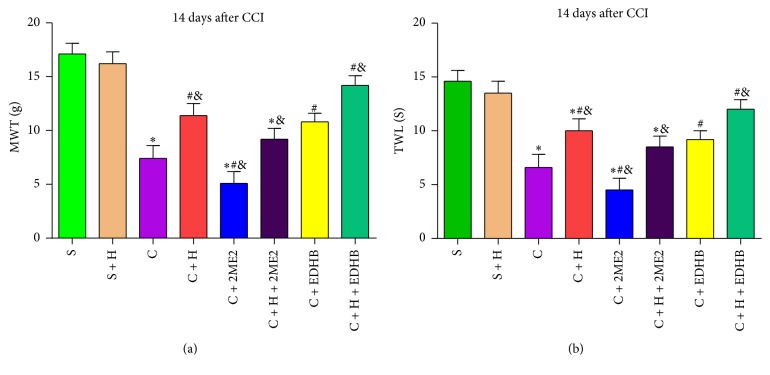
*Effect of EDHB and HRS on thermal hypersensitivity and mechanical hypersensitivity at 14 days after CCI.* (a) Mechanical withdrawal threshold. (b) Thermal withdrawal latency. Data are expressed as mean ± SD; ^*∗*^*P* < 0.05 versus sham; ^#^*P* < 0.05 versus CCI; ^&^*P* < 0.05 versus CCI + HRS.

To investigate the role of HIF-1*α* in the HRS treatment of NP, we used the HIF-1*α* inducer EDHB and HIF-1*α* inhibitor 2ME2 in the subsequent experiment. Compared with the S and S + H group, group C had reduced MWT and TWL values. As compared with group C, group C + 2ME2 reduced the MWT and TWL values at 14 days; in groups C + H and C + EDHB, the MWT was improved and the TWL was prolonged at 14 days. In contrast to group C + H, group C + H + 2ME2 reduced the MWT and decreased the TWL (*P* < 0.05, *n* = 12, one-way ANOVA). Group C + H + EDHB prolonged the TWL and increased the MWT (*P* < 0.05, *n* = 12, one-way ANOVA; Figures 5(a) and 5(b)).

### 3.8. HIF-1*α* Inducer EDHB and HRS Activate the Autophagy Procedure in Rats with CCI of the Spinal Cord

**Figure 6:**
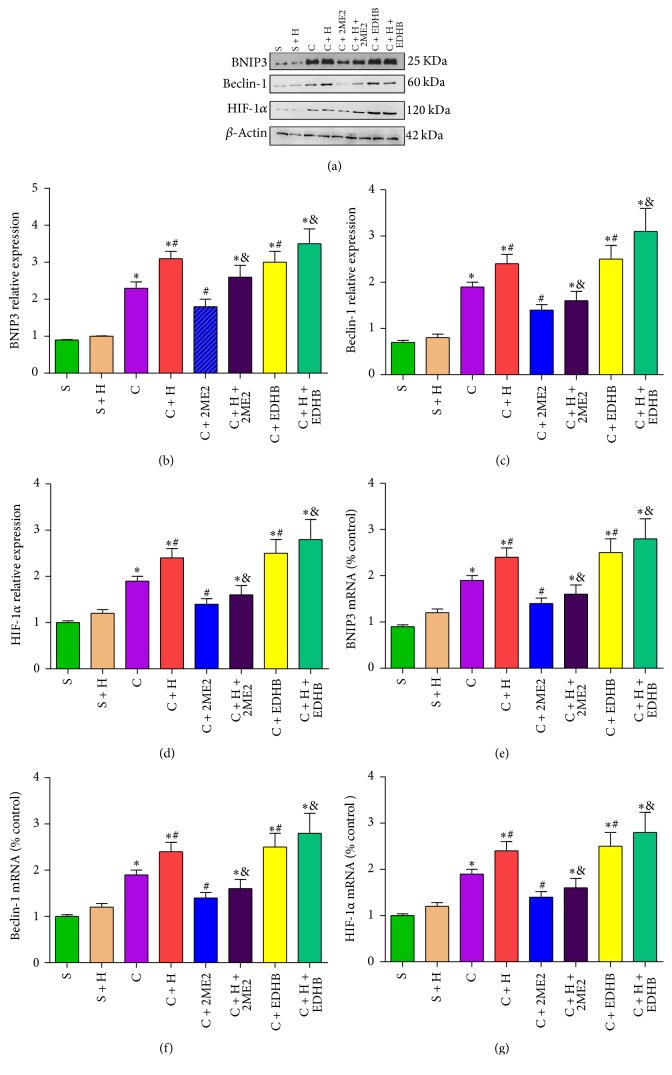
*The expression of BNIP3, Beclin-1, HIF-1α protein, and mRNA after EDHB and HRS treatment.* **(**(a)–(d)) The expression of BNIP3, Beclin-1, and HIF-1*α* protein in spinal cord at 14 days. ((e)–(g)) The mRNA expression of BNIP3, Beclin-1, and HIF-1*α* in spinal cord at 14 days. Data are expressed as mean ± SD; ^*∗*^*P* < 0.05 versus sham; ^#^*P* < 0.05 versus CCI. ^&^*P* < 0.05 versus CCI + HRS.

Compared with group S, the Beclin-1, HIF-1*α*, and BNIP3 in the spinal cord of group C, group C + H, group C + H + 2Me2, group C + EDHB, and group C + H + EDHB were significantly increased on the 14th postoperative day (*P* < 0.05, *n* = 12, one-way ANOVA). Compared with group C, the expression of Beclin-1, HIF-1*α*, and BNIP3 in the spinal cord was upregulated 14 days after nerve ligation in group C + H, group C + EDHB, and group C + H + EDHB (*P* < 0.05, *n* = 12, one-way ANOVA; Figures 6(a)–6(g)) and downregulated in group C + 2Me2 (*P* < 0.05, *n* = 12, one-way ANOVA). Compared with group C + HRS, the expression of Beclin-1, HIF-1*α*, and BNIP3 was upregulated in the spinal cord at 14 days after nerve ligation in group C + H + EDHB and downregulated in group C + H + 2Me2 (*P* < 0.05, *n* = 12, one-way ANOVA; Figures 6(a)–6(g)). Thus, the HIF-1*α* inducer EDHB and HRS activate autophagy in rats with CCI of the spinal cord.

### 3.9. HIF-1*α* Inducer EDHB and HRS Increases Autophagosomes and Autolysosomes in Rats with CCI of the Spinal Cord

**Figure 7:**
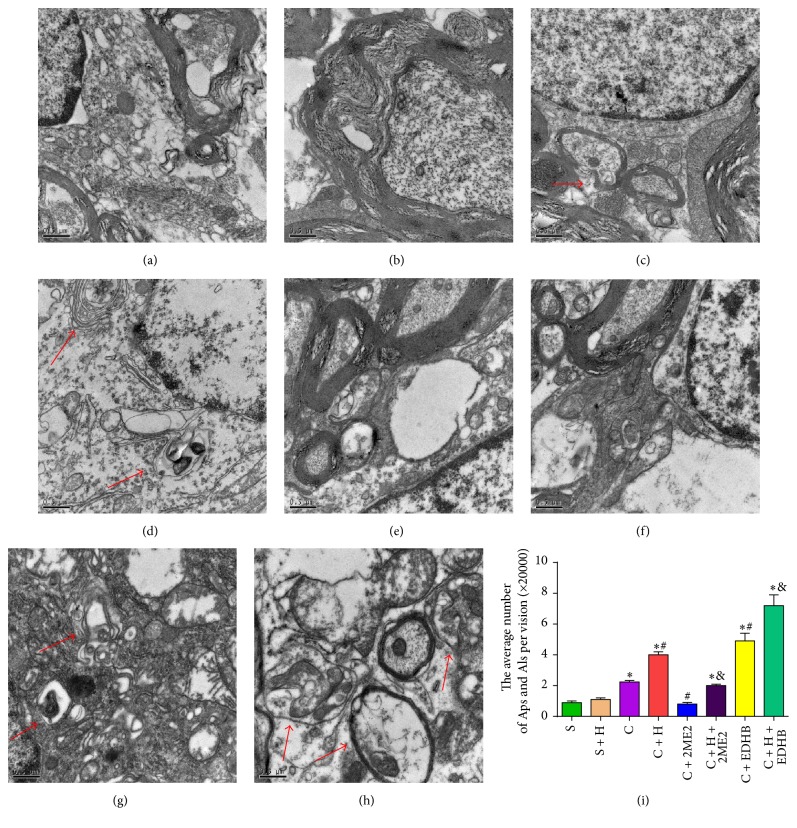
*Autophagosomes (Aps) and autolysosomes (Als) in the eight group's spinal cord neurons were evaluated with TEM.* (a) Sham group. (b) Sham + HRS group. (c) CCI group. (d) CCI + HRS group. (e) CCI + 2ME2 group. (f) CCI + HRS + 2ME2 group. (g) CCI + EDHB group. (h) CCI + HRS + EDHB group. The number of autophagosomes (Aps) and autolysosomes (Als) in the spinal cord; ^*∗*^*P* < 0.05 versus sham; ^#^*P* < 0.05 versus CCI; ^&^*P* < 0.05 versus CCI + HRS. One autophagosome has engulfed a mitochondrion (arrow). Scale Bar = 500 nm.

The number of autophagosomes and autolysosomes was evaluated with TEM. The number of autophagosomes and autolysosomes in group C, group C + H, group C + H + 2Me2, group C + EDHB, and group C + H + EDHB was significantly increased on the 14th postoperative day compared with that in sham-operated rats (*P* < 0.05). Compared with group C and group S, HRS and EDHB injection significantly increased the number of autophagosomes and autolysosomes in the spinal cord at 14 days after CCI (Figures 7(d), 7(g), and 7(h)), whereas administration of 2Me2 significantly reduced the number of autophagosomes and autolysosomes in group C + 2Me2 and group C + H + 2Me2 (Figures 7(e) and 7(f)).

## 4. Discussion

In the present study, we found that HRS has a potent analgesic effect against NP and reduces mechanical hyperalgesia and allodynia and activation of cell autophagy in neuropathic pain through the HIF-1 pathway, as confirmed by western blot, RT-PCR, and TEM.

NP is a chronic pain state due to peripheral or central nervous system lesions and is characterized by spontaneous pain, hyperalgesia, and pain hypersensitivity; causes include trauma, infection, nerve toxicity drugs, tumor, autoimmune diseases, and vitamin deficiency [32]. We used CCI rats as the NP animal model, which was first reported by Bennett and Xie in 1988 [26] to simulate the basic features of clinical NP. In the present study, CCI rats in the experimental group pulled the left hind leg away, toes together, foot dangling, and taking on a resting position towards the healthy side. Sometimes the rats licked the left hind leg or showed claudication and other protective behavior, which suggest that spontaneous pain occurs after CCI. Quantitative evaluation of animal hyperalgesia and allodynia mainly relies on MWT and TWL. We found that MWT and TWL started to decline on postoperative day 3, peaked on postoperative day 7, and was maintained for the remainder of the 14 days. Our results were consistent with previous findings on CCI-induced allodynia [33, 34]. We successfully established the CCI model; rats in group S showed no responses to mechanical or heat stimulation.

Hydrogen is the lightest element in the periodic table of elements; it is one of the simplest and abundant elements in nature. In basic and clinical research studies, the method for administration of hydrogen mainly included inhalation of hydrogen gas [6], drinking hydrogen water [35], injection of HRS, and hydrogen-loaded eye drops [36]. HRS has some advantages, such as simple preparation, high safety, and being easy to carry; there is no difference between this treatment effect and that of direct inhalation of hydrogen, and it is suitable for the research of chronic diseases [37]. The concentration of drinking HRS cannot be controlled; thus, injection of HRS can more strictly control and ensure the concentration of hydrogen [38]. As in our previous study, this experiment adopted intraperitoneal HRS injection of 10 mL/kg daily, to study the therapeutic effects of HRS in NP [8, 9]. Our results are consistent with previous research results [8, 9]. HRS treatment could alleviate allodynia and hyperalgesia behavior.

Autophagy is an intracellular catabolic process ubiquitously in eukaryotes. Autophagy contributes to cell repair and reconstruction and provides energy and raw materials for the degradation of damaged cytoplasmic constituents and organelles in the lysosomes [39]. Under normal circumstances, autophagy is in steady state and occurs at low level. When there are no factors conducive to cell survival, such as ischemia, hypoxia, and nutrients, autophagy can be induced to protect and maintain the homeostasis. There are defects in the autophagy machinery in NP [15]. Our study revealed that upregulation of autophagy can decrease the development of NP in HRS-treated CCI rats. When autophagy was pharmacologically inhibited by 3-MA, the neuropathic condition worsened markedly. It is not clear how upregulation of autophagy can benefit NP. Upregulation of autophagy by RM and other small molecules has been shown to suppress aggregation of disease-linked proteins, including huntingtin and alpha-synuclein, and to reduce cellular toxicity and myelination in explant cultures from neuropathic mice [40]. Previous studies have reported that impaired autophagy can affect the function of spinal GABAergic interneurons [41], spinal microglia [42], Schwann cells [43], and dorsal root ganglion neurons [44], which are pain transmission elements and express a series of proinflammatory cytokines. These not only are related to hyperalgesia and allodynia but are also involved in the induction and development of NP [42]. Autophagy activity in Schwann cells may be a powerful pharmacological approach for preventing the onset and chronification of NP in clinical inflammatory responses [43]. When autophagy was activated by inflammatory signals, the levels of proinflammatory cytokine secretion were regulated and controlled; for instance, IL-1 production was limited [45]. We cannot rule out the possibility that the antinociceptive effects of HRS could be attributed to the role of anti-inflammatory properties in autophagy. A recent study reported that autophagy is a double-edged sword and has different influences on the progression of the disease and excessive autophagy may cause damage in some diseases [46]. We believe that autophagy may be induced naturally by hydrogen, but further studies are required to determine how the levels are controlled.

We all know that ischemia and hypoxia are thought to play an important role in NP conditions [47]. HIF-1*α* can initiate the transcription of an array of target genes that are vital for cellular adaption to hypoxia [48]. Recent reports have also indicated that HIF-1*α* may induce autophagy under hypoxic conditions via different signaling pathways, leading to the transcription of the target gene, BNIP3, which competes with Bcl-2 and Bcl-XL for interaction with Beclin-1 to induce autophagy [23]. BNIP3 is also known as a hypoxia-responsive protein, leading to autophagy, which may promote both tumor suppression and tumor growth [49]. Our results showed that upregulated HIF-1*α* activation has been found in group C, whereas HRS induced increased HIF-1*α* expression in the spinal cords of rats in group C. HIF-1*α* inducer EDHB and HRS activate the autophagy procedure and increase the number of autophagosomes and autolysosomes and unregulated BNIP3 in rats with CCI of the spinal cord. These results suggest that HRS activated more HIF-1*α* and target genes to activation of autophagy in a mouse model of CCI.

Prior studies have shown that HIF-1*α* is protective in terms of the pain caused by acute heat and cold, but it may promote the development of NP in case of ongoing activation in injured neurons [50]. It seems that HIF-1*α* plays a dual role in pain regulation in NP. We suspect that autophagy increased by HIF-1*α* may be associated with the optimal treatment time in HRS-treated NP, and the application of HRS as early as possible may be more advantageous to therapy.

How HRS activates the HIF-1*α* pathway to induce autophagy remains unclear. There are two ways of enhancing the activity of HIF-1*α*, either to enhance the synthesis of HIF-1*α*, such as gene transfer or to inhibit the degradation of HIF-1*α* [51]. Subsequent studies have indicated that the anti-inflammatory effect of hydrogen may activate Nrf2 [51] and its downstream target, heme oxygenase-1 (HO-1), in different experimental models [52]. Previous studies have indicated that HO-1 can trigger and regulate HIF-1*α* expression in hypoxic tumor cells [53]. Our previous study indicated that HRS was involved in the activation of HO-1/CO signaling during NP in rats [9]. These molecules are in the upstream pathways of HIF-1*α*, which are also autophagy-related pathways. We predict that HRS treatments in this study increased HIF-1*α* activity and may be connected with upstream pathways.

In summary, the present data suggested that HRS reduces mechanical hyperalgesia and activation of cell autophagy in NP via HIF-1-dependent pathways. This finding could explain the mechanism treatment of NP by HRS, at least in part.

## Figures and Tables

**Table 1:** Primers used for the gene expression analysis.

Genes	Forward	Reverse
HIF-1*α*	5′-TGCTCATCAGTTGTTGACTT-3′	5′-TGGGCCATTTCTGTGTGTAA-3′
Beclin-1	5′-TTTCAGACTGGGTCGCTTGC-3′	5′-CTTTTGTCCACTGCTCCTCCG-3′
P62	5′-CGTCTGCCCAGACTACGACT-3′	5′-GTGTCCGTGTTTCACCTTCC-3′
GAPDH	5′-CGGAGTCAACGGATTTGGTCGTAT-3′	5′-AGCCTTCTCCATGGTGGTGAAGAC-3′

## Abbreviations

HRS: Hydrogen-rich saline
CCI: Chronic constriction injury
MWT: Mechanical withdrawal threshold
TWL: Thermal withdrawal latency
HIF-1*α*: Hypoxia-inducible factor-1*α*
NP: Neuropathic pain
mTOR: The mammalian target of rapamycin
3-MA: 3-Methyladenine
2ME2: 2-Methoxyestradiol
EDHB: Ethyl-3,4-dihydroxybenzoate
BNIP3: Bcl-2/adenovirus E1B-19 kDa-interacting protein 3
P62: SQSTM1/p62
GAPDH: Glyceraldehyde-3-phosphate dehydrogenase.
